# How deeply should we analyze non-covalent interactions?

**DOI:** 10.1007/s00894-023-05460-4

**Published:** 2023-02-09

**Authors:** Timothy Clark

**Affiliations:** Computer-Chemistry-Center, Department of Chemistry and Pharmacy, Friedrich-Alexander-University Erlangen-Nuernberg, Naegelsbachstrasse 25, 91052 Erlangen, Germany

**Keywords:** Polarization, Covalent, Charge transfer, Bonding analysis

## Abstract

**Context:**

Just how much effort and detail should we invest in analyzing interactions of the order of 5 kcal mol^−1^? This comment attempts to provide a conciliatory overview of what is often a contentious field and to pose some questions that I hope will eventually lead at least to some consensus.

**Methods:**

This is an opinion article without calculations or data.

## Introduction

This comment contains my reaction to, and my questions that arise from, the *Journal of Molecular Modeling Conversation* on *Non-Covalent Interactions* [1–4]. To be honest, I never intended to enter the business of analyzing bonding interactions, but our 2017 paper on the σ-hole [5] pitched me into a discussion that I had not expected and in which I did not really wish to participate. As I write this, the original σ-hole paper has been cited more than 1700 times and has generated rivalling groups of followers and critics. I hope with this contribution to pose some relevant questions and perhaps in some cases achieve unity. I think that the effort to generate a consensus view of non-covalent interactions is worthwhile, even if likely doomed to failure. In the following, I will touch on some salient points and hope to initiate a fruitful discussion. A central point is to distinguish between chemical models, which are the essence of how we think about chemistry, and “physical reality,” which I have put in quotation marks. Martin Hicks and I have considered the roles of models in chemistry in more detail in a recent essay [6]. In short, the majority of the concepts we use in chemistry go back to some sort of chemical model, usually a bonding model. This fact alone should preclude heated discussions about the merit of competing models (as long as they work): Given the dependence of chemical thinking on models, controversial arguments about which model is best suited cannot really be regarded as contributions to productive science.

A particularly frustrating phenomenon is that authors often incorrectly or incompletely use a “competing” model to their own in order to demonstrate that it fails for a given problem. Unfortunately, in many cases, our peer-reviewing system fails to catch work of this type, even in “respectable” journals. There are some failures of common theories, such as for instance the lack of a universal connection between bond critical points and bonding interactions in QTAIM [7], or the wildly different assignment of “polarization” and “covalent” energy contributions for the water dimer in different energy-decomposition analyses [8], so that these features of the model in question should no longer be used, or used with caution. However, in general, most complete model analyses give the right answer for intermolecular interactions.

A disturbing feature of the current situation is that many papers are published that only provide an analysis (i.e., a theoretical/computational chemical interpretation), within a given model, of an intermolecular bonding interaction. The situation is made more complex by the fact that such interpretations are often based on the pioneering work of Roald Hofmann, Kenichi Fukui, and many others that provided an, at that stage, unknown explanation of bonding or reactivity phenomena and provided the tools for a new level of prediction in chemistry.

Paul Popelier: *Ironically, Roald Hoffmann is not helping to clean up the unsatisfactory situation that the community finds itself in, in which alternative methods make contradictory predictions. Such methods can only coexist (by the principle of falsification) if they make the same predictions, such as wave mechanics and matrix mechanics. Bohrian and Newtonian mechanics need to be discarded when applied in the quantum world (at all or beyond H). According to Hoffmann’s philosophy the latter would be OK, even enriching physics! Surely, to mention another example, the “opinion” that F*=*m/a instead of the correct F*=*ma does not enrich Newtonian mechanics, does it?*

I find the completeness of a model important: I do not believe that it is correct to assign the missing portion of the binding energy of a complex within a model to a specific effect (e.g., charge transfer, CT). This practice means that any deficiency in calculating one or more of the other terms will be assigned to the one not calculated. I much prefer models such as SAPT that calculate the entire complexation energy, not least because the result can be compared with high-level calculations.

Paul Popelier: *Well, I must congratulate you for the candor of this Introduction. Rarely do I see such a glimpse of behind-the-screens turmoil or a sociological essay on the tribe called “interpretative quantum chemists”. You are succeeding in stirring things up, and echoing complaints I have voiced in some of my own book chapters.*

Tim Clark: *Yes, Paul, emotions often run high in fields that are really a matter of interpretation. Martin Hicks and I have tried to emphasize the model nature of chemical thinking, *[6]* but with little resonance. Competing models take on religious traits. I suspect that much of the criticism of QTAIM is aimed at its conclusions, not at the technique itself, which is uniquely defined.*

Paul Popelier: *Richard Bader’s ways have often raised the temperature of debates unnecessarily. This infelicity has perhaps added to alienating members of the Theoretical and Computational Establishment and slowing down QTAIM’s uptake. Of course, computational demand of the quantum topological energy decomposition method IQA and its algorithmic complexity have not helped either.*

In the following, I have some questions for the authors of the conversation:

1. *Charge transfer vs polarization*

The elephant in the room is almost always distinguishing between polarization and CT, which is sometimes called donor–acceptor interaction or covalent contribution. Here, we need to distinguish between valence bond (VB) and most molecular orbital (MO) theories. As pointed out by Mo et al., [2] charge transfer is actually defined in VB theory as the contribution of structures in which an electron has been transferred from one chemical moiety to another, where the two are not connected by a bond. This is a clear definition, even though the results depend on the definition and optimization of the orbitals used to define the individual resonance structures. Thus, VB theory does offer a definition of charge transfer, so that, with sufficient definition of the exact calculational techniques used, CT can be discussed within the VB model. Interestingly, Mulliken’s original discussion of CT occurred within a VB context [9]. Molecular orbital theory is not as lucky: Polarization and CT cannot be separated uniquely in calculations on intermolecular complexes, as has been pointed out many times [6, 10]. QTAIM is in a unique position in this respect because it provides a unique definition of the separating surface between atoms, so that “atomistic” concepts such as CT are also well-defined. It seems to me that the sum of polarization and CT is likely to be constant for different analyses of a given calculation. Indeed, Sokalski and Roszak [11] and Stone [12] have shown that the proportion of CT relative to polarization decreases with increasing size of the basis set, and eventually disappears. Surely, what we call this term is purely a question of semantics unless atoms can be defined uniquely. It is essentially exclusively called CT in NBO analyses, which cannot distinguish uniquely between CT and polarization [13].

Paul Popelier: *I believe that there is no elephant within the QCT framework. According to the QTAIM view, CT and polarization are neatly separated. My article (which is part of this Conversation) discusses this point on page 13 at the bottom right, for example, but also elsewhere in that article. There is also more on this view in *[14]*.*

*Secondly, possibly the “crispest” work on this matter was carried out in the Brazilian group of Roy Burns. *[15]* Importantly, their so-called CCTDP model is connected to the EXPERIMENT of infrared. It can judge population analyses by how well they are able to connect to experiment. It turns out that QTAIM comes out really well compared to DDEC6 (and variants), Hirshfeld, CHELP and GAPT (but with a twist).*

2. *Calculating polarization*

Point-charge models have been used with great success by, for instance, Kersti Hermannson, [16], Tore Brinck [17], and ourselves to illustrate the effects of polarization (neglecting exchange). In one case [18], we used a large lattice array of point charges to represent the electron density of the second molecule in the complex and iterated to self-consistence. In another case, the polarization effect was found to be linked to an “anomeric” geometry change in which the strength of the anomeric effect is increased (leading to a corresponding change in geometry) by a polarizing field [19]. Such calculations have repeatedly shown the effects of classical polarization, but the resistance to using them is very high. Although the calculations are very simple and can be performed with almost every ab initio or DFT package, in only one case have authors whose papers I have refereed ever taken up the suggestion to carry them out. Relatively recently, Head-Gordon and coworkers [20] have analyzed NBO, constrained DFT, regularized SAPT, and various versions of ALMO and essentially conclude that ALMO results are closer to traditional ideas of charge transfer than constrained DFT (CDFT). They attribute differences, which can be extreme, to the definition of the reference “CT-free” state. Most disturbingly, they report CT energies for the water dimer between − 9.17 (NBO) and − 0.36 kcal mol^−1^ (CDFT).

Tim Clark: *Can any of the available ab initio/DFT programs calculate a molecule in the electric field of another, either directly or using a distributed multipole model? Would it not be useful to use such calculations (with geometry optimization) to compare the polarization energies thus obtained with those from, for instance, SAPT or ALMO?*

Yirong Mo: *Yes, the block-localized wavefunction (BLW) method can precisely do this job and generate the so-called “in situ” orbitals where MOs of each monomer are perturbed by other monomer(s), but there are no electron transfers among monomers. ALMO can do the same job as it is identical to the BLW method.*

Tore Brinck: *That should be possible to calculate using the FMO-module of Gamess.*

3. *Dispersion*

Both the London [21] and Feynman [22, 23] dispersion models involve a small shift of electron density compared to the uncorrelated wavefunction or electron density. This shift is not reproduced by dispersion corrections that use classical potentials to add dispersion, such as those usually used for DFT.

Tim Clark: *Is it correct to use wavefunctions that do not contain the shift in electron density caused by dispersion to analyze weak intermolecular interactions? (e.g. are DFT-D3 calculations of weakly bound complexes at all suitable for analysis by density-based techniques?)*

Yirong Mo: *Computationally it is achievable to derive dispersion-corrected electron density if the corrected energy is used as the final energy. Besides, there are also density-based dispersion corrections (D3 is not).*

Tore Brinck: *It depends on the purpose of the analysis. If the goal is to get accurate energies, it is well known that DFT-D3 in most cases works very well. On the other hand, if the main goal is understanding, the method may be less appropriate. Still it should be remembered, that there are very few, if any, methods that allow for separate characterization of the shift in electron density due to dispersion.*

Paul Popelier: *For the purpose of analyzing any interatomic interaction (whether intermolecular or intramolecular) we prefer to use non-DFT, post-Hartree-Fock wavefunctions. In a recent mini-review *[24]* we show how a quantum topological energy decomposition method known as IQA extracts chemical insight from MPn (n*=*2,3,4) wavefunctions, even at intra-atomic level. Such an analysis involves an invariably gigantic two-particle density matrix, which in our more recent work on CCSD wavefunctions *[25]* revealed sharp patterns of transferability of correlation energy in water clusters.*

Tim Clark: *Is it not problematic to define the dispersion contribution as that of the classical correction potential? This has, among other factors, been parametrized to compensate for deficiencies in the internuclear repulsion?*

Yirong MO: *I assume that the author is talking about the dispersion correction not the total dispersion contribution. D3 is the “dispersion correction” and the lion’s share of dispersion energy is imbedded in the correlation functional, whose energy contribution can be extracted.*

Tim Clark: *This is true but my question is about what the classical potential is actually correcting because the basic functional may give wildly wrong repulsion-energy curves that must be corrected.*

Tore Brinck: *Yes, it is not recommended, in particular when using DFT-functionals, such as the Minnesota functionals, which are parameterized to account for short to mid-range dispersion. However, to define dispersion as the difference between the MP2 interaction energy and HF interaction energy is not that “smart” either, as other components of the interaction energy, such as electrostatics, also depend on electron correlation.*

Paul Popelier: *Yes, there are two vulnerabilities in defining dispersion like that. Firstly, the concept of dispersion is locked into (typically long-range) perturbation theory, which means that intramolecular (let alone intra-atomic) dispersion does not exist. Secondly, the classical inverse sixth power (which is theoretically justifiable in perturbation theory) may not capture the behaviour of electron correlation (which gives rise to dispersion) at short or medium range. Some of our hitherto unpublished work discovered the power law obeyed by the IQA post-Hartree-Fock interatomic electron correlation energies. Note that this approach does not introduce damping functions. Moreover, it is cleanly separated from interatomic repulsion energy, which has been successfully fitted to a Buckingham-type potential, thereby confirming its steric nature *[26]*.*

4. *Partitioning energy terms*

Perhaps, the most problematic area of analyzing noncovalent interactions is the partitioning of the total binding energy into notionally orthogonal contributions from different physical effects. This is an ultimately unreasonable process because, as Hurley [27–30] and Bader [31] demonstrated as long ago as the 1950s, once the correct variational electron density is available, the electronic potential energy can be calculated from Coulomb’s law alone. Thus, the zealot view of intermolecular interaction energies is that they are 100% electrostatic. This is often not appreciated and does not, in any case, provide a viable technique for calculating molecular energies because of the stringent requirements for completely variational structures and electron densities (including the basis set) outlined by Hurley [27]. Nonetheless, purely electrostatic calculations can provide information, if not chemically accurate potential energies [32].

Thus, the different notional contributions to the bonding energy actually reduce to the effect of the given interaction type on the electron density. Nonetheless, physically founded perturbational techniques to calculate the total interaction energy, as in SAPT [4], can provide insight into the origins of the stabilizing effects. Here, it is important that all contributions are calculated and that their sum agrees with (or is more accurate than) the originally calculated energy (it is possible, for instance, to obtain CCSD(t)-quality interaction energies from DFT densities [4]). Calculating “all but one” interactions and attributing the missing energy to the neglected interaction is fraught with difficulties. It is interesting to note here that many authors have attributed great importance to exchange repulsion, which is available via SAPT. There are many studies in the literature in which a large positive exchange repulsion term is slightly overbalanced by an attractive electrostatic/induction term. I prefer to consider the (small) sum of these two large terms because repulsion will increase until the minimum is reached, which depends on the attractive terms.

Tim Clark: *How much detail is sensible in partitioning small (ca. 5 kcal mol*^*−1*^*) interaction energies? To put this number into perspective, positive and negative charges of approximately 0.17 e*^*−*^* at a distance of 2 Å give this stabilization.*

Paul Popelier: *Interactions of the order of 20 kJ mol*^*-1*^* (a typical medium-strength hydrogen bond) should not be ignored, nor seen as irreducible.*

*For example, our analysis of electron correlation patterns in molecules and complexes shows that there is substantial dispersion energy between non-bonded hydrogen atoms, and that this H…H interaction is always negative (see Table 3 in reference *[24]* for a possible range of −0.3 kJ mol*^*−1*^* to −4.9 kJ mol*^*−1*^*). This observation highlights the importance of the oft neglected role of intramolecular interatomic dispersion in the stabilization of systems. In fact, these H…H dispersive interactions typically occur multiple times and, when added, can lead to non-negligible energies. Some years ago, H…H dispersive interactions have been reported for hydrocarbons *[33]* in connection with protobranching, which is the effect of enhanced stability of branched alkanes.*

Yirong MO: *EDAs are somewhat arbitrary but can provide illuminating insights (for the examples of benzene with NO*^+^ *and NO*_*2*_^+^*, their binding energies are similar but chemical reactivity is dramatically different). SAPT can get accurate data but the connection between math formula to the physical interpretation is loose in my opinion. I think that the use of multiple methods (e.g., including the results from the charge model) may generate more convergent conclusion.*

Tore Brinck: *Still a non-covalent interaction of 5 kcal mol*^*−1*^* is considered relatively strong. Non-covalent interactions often work in concert, such as in proteins or DNA, and even small individual energy contributions may after summation play a significant role. As an example, it is today generally accepted that dispersion plays a crucial role for the stabilization of the DNA-helix.*

Tim Clark: *Some interactions, such as charge-transfer and polarization are generally inseparable within MO theory. Why are myriads of manuscripts published that claim dominance of one over the other?*

Paul Popelier: *This problem does not occur within the framework of Quantum Chemical Topology (QCT), which is a real-space alternative to the still traditional Hilbert-space interpretations. Hence, I can only guess why such myriad of manuscripts exists, that is, by the general way the community of interpretational quantum chemistry operates. This community is not good at abandoning misleading or even wrong concepts. In contrast, in other parts of Science, concepts are abandoned such as phlogiston for example. In the absence of falsification, which should irreversibly progresses Science (even cosmology), interpretational quantum chemistry allows conflicting methods to coexist. However, it is acceptable for alternative methods to coexist, but only if they make the same predictions. For example, wave mechanics, matrix mechanics and Bohmian quantum mechanics can all coexist but Bohrian quantum mechanics not (if one looks beyond the hydrogen atom).*

Yirong Mo: *In my opinion (as discussed in our contribution “The roles of charge transfer and polarization in non‑covalent interactions: a perspective from ab initio valence bond methods” with Sason and David), there is obvious difference in their physical origins.*

Answer: *Yes, this is true for VB theory, as I point out. However, there are only a few limiting examples (infinite separation, structural symmetry) for MO theory*.

Tore Brinck: *That is a good question. It may partly be connected to the quest for distinguishing between covalent and non-covalent interactions. However, it is questionable if covalency should be set equal to charge transfer. As an example, a strong dative bond clearly has a significant covalent contribution, but the electron pair is not really donated from the donor to the acceptor but rather shared between them.*

Tim Clark: *I submit that the partition between charge transfer, however defined, and polarization has no practical consequences. The same is true of separate (exchange) repulsion and induction/electrostatic terms in SAPT. Would it not be simpler and more consistent to lump CT and polarization and induction and (exchange) repulsion into one term each?*

Paul Popelier: *Again, within QCT, there is no need to lump any energy terms. Charge transfer is well separated from dipolar polarization, and discussing them separately is meaningful. Some time ago *[34]* an IQA study on standard hydrogen-bonded complexes found it useful to combine IQA’s (intra-atomic) deformation energy (E*_*def*_*) with (interatomic) exchange energy (V*_*x*_*) into an exchange-repulsion energy (XRC). This was mainly done for comparison purposes rather than to define an instrumental type of energy. It was found that the IQA exchange-repulsion was very small, considerably below the usual values found with other energy decomposition schemes.*

Yirong MO: *In ADF, CT and polarization are indistinguishable and the sum is the “orbital interaction” energy.*

Tore Brinck: *In general, it is sound to not differentiate between CT and polarization/induction. Exchange repulsion, on the other hand, is mainly considered a first order interaction term, and therefore often combined with electrostatics. However, changes in the electron density of the monomers due to polarization and CT are likely to have an effect on the repulsive potential. It is not clear if that equals to the exchange-induction cross-terms in SAPT.*

## The title question

My core question is one of publication ethics, rather than science:

Tim Clark: *What is the justification for articles that only analyze intermolecular interactions within a given (or even using several) bonding model(s)? Generally, they do not draw any chemical conclusions that lead either to new knowledge or a predictive model of the given interaction for new systems. How do they contribute to science as a whole? Are they justified, given the exploding number of publications?*

Paul Popelier: *As editor of a book in Elsevier’s planned collection of books entitled Comprehensive Computational Chemistry (to be published in 2023 hopefully) I have brought together 18 chapters, each on a specialist topic in interpretational quantum chemistry. This content is impressive, varied and technically sophisticated, often accompanied by well-tested and mature computer programs. However, it appears that this magnificent body of work is still not able to settle for unique explanations behind chemical phenomena. However, it is very important that this community can extract consistent, unambiguous and non-contradictory concepts and rules from modern wave functions. Currently many old textbook explanations remain unchallenged, even if teachers continue to feel uncomfortable teaching them, for decades, because deep down they know that something is not quite right. Trustworthy rules need to guide experimentalists in their future molecular designs.*

Tim Clark: *There are related questions, such as whether we should be aiming for as general a bonding model as possible or whether local, specific models should be proposed for every subtype. Above all, is it legitimate (also for experimentalists) to criticize a given model on the basis of the authors’ expectations without ever having tested the model adequately?*

*I obviously have opinions and preferred answers to these questions. It seems to me that testable models should be tested, and untestable ones should be considered speculative. I have also always considered it important to have a single general explanation for related phenomena. I imagine that it would be a nightmare to teach hydrogen, halogen, pnictogen, and tetrel bonding in any cogent and consistent way without pointing out that they are all variations of the same theme. Why, then, do we need all these designations?*

Yirong Mo: *Inflation is everywhere, not only in the study of intermolecular interactions. There are many tiers for journals and thus for articles.*

Tore Brinck: *No, the goal of science should always be to increase our understanding.*

Paul Popelier: *Yes, we should aim for a general model. However, let us not forget that Science (and also mathematics) progresses from the special to the general. For example, whereas one generation of budding chemists had to learn a disparate bunch of substitution reactions in organic chemistry, the next generation learnt about S*_*N*_^*2*^* reactions, which introduce more abstract concepts such as nucleophile and leaving group. Another spectacular example is that of Maxwell’s equations. Instead of his original list of 20 odd equations, contemporary textbooks quote the familiar 4 equations, while geometrical algebra reduces even those equations to only one.*

There are related questions, such as whether we should be aiming for as general a bonding model as possible or whether local, specific models should be proposed for every subtype. Above all, is it legitimate (also for experimentalists) to criticize a given model on the basis of the authors’ expectations without ever having tested the model adequately?

I obviously have opinions and preferred answers to these questions. It seems to me that testable models should be tested, and untestable ones should be considered speculative. I have also always considered it important to have a single general explanation for related phenomena. I imagine that it would be a nightmare to teach hydrogen, halogen, pnictogen, and tetrel bonding in any cogent and consistent way without pointing out that they are all variations of the same theme. Why, then, do we need all these designations?

Tim Clark: *Is the proliferation of “X-bonding” definitions not detrimental, especially for teaching? Should we not be aiming at a general model?*

Yirong Mo: *I agree with the author that the definitions of various X-bonding are unnecessary and confusing. Non-covalent bonding is enough to include all types of interactions.*

Tore Brinck: *I do not think that it is detrimental in itself to teach the students about hydrogen and halogen bonds, as they are to a great extent unique in their characters compared to other non-covalent interactions. After all, one cannot overestimate the importance of understanding hydrogen bonds in areas such as drug design. The problem is that many text books, even at advanced level, give the impression that they have a different physical origin than other non-covalent interactions.*

Tim Clark: *Should it not be a criterion for acceptance of manuscripts that criticize “competing” analysis techniques that they demonstrate conclusively with the full model being criticized that it indeed fails?*

Yirong Mo: *I was once “lectured” by a journal editor that I should focus on my data and my interpretation, rather than criticize any other analysis techniques. But it is certainly very important to examine various methods together as each method has its own merits and fails.*

Tore Brinck: *In principle it seems like a good idea, but in practice it may be hard to implement. To start with, the journals implementing it would have to define what they mean by “conclusively”.*

Paul Popelier: *I have never been an editor but I wish anyone who is good luck with that prospect. In an ideal world this would help scientific progress though.*

## Conclusions

Readers can certainly discern my preferences from the text above, and from the questions that I have asked, but I am genuinely interested in answers to the above questions. I emphasize, however, that I consider our ultimate goal to be providing predictive interpretations and analyses of non-covalent interactions. I would like to be as model-agnostic as possible. I once, for instance, criticized the misuse of bond critical points in some published analyses [7]. Nevertheless, I recognize that QTAIM can make very valuable contributions, for instance in the difficult area of translating QM calculations to atomistic approaches such as force fields, and that it is one of the very few fields that provides a unique partitioning between atoms. Similarly, my group has used DFT-SAPT quite extensively simply because it has the ability to predict interaction energies at a very high level for systems too large for definitive calculations. In this case, SAPT provides the (numerical) answers that we need.

Above all, I believe that we theoreticians have a duty to be as objective as possible because what we publish has an effect: We should never forget that published bonding analyses strongly influence experimentalists, who may become disciples to the exclusion of alternative models and interpretations.

It is perhaps symptomatic that controversial arguments often center on inseparable types of interactions, so that, in the end effect, the outcome of the controversy has no “real” consequences.
